# Expression of ALG3 in Hepatocellular Carcinoma and Its Clinical Implication

**DOI:** 10.3389/fmolb.2022.816102

**Published:** 2022-06-15

**Authors:** Zhen Zhao, Zehao Zheng, Jianfeng Huang, Jianxi Wang, Tianyi Peng, Ye Lin, Zhixiang Jian

**Affiliations:** ^1^ School of Medicine, South China University of Technology, Guangzhou, China; ^2^ Department of General Surgery, Guangdong Provincial People’s Hospital, Guangdong Academy of Medical Sciences, Guangzhou, China; ^3^ Shantou University of Medical College, Shantou, China; ^4^ The Second School of Clinical Medicine, Southern Medical University, Guangzhou, China

**Keywords:** ALG3, hepatocellular carcinoma, immune infiltration, prognosis, clinical implication

## Abstract

**Background:** Recent studies have shown that alpha-1,3-mannosyltransferase (ALG3) promoted tumorigenesis and progression in multiple cancer types. Our study planned to explore the clinical implication and potential function of ALG3 in hepatocellular carcinoma.

**Materials and Methods:** Data from public databases were used to analyze the ALG3 expression and its impact on the clinical significance of patients with HCC. The ALG3 expression was confirmed by qRT-PCR and Western blot. Immunohistochemistry was used to confirm the ALG3 expression and explore its clinical implication in HCC. KEGG, GO, and GSEA enrichment analyses were utilized to explore the biological pathways related to ALG3 in HCC. TIMER2.0 was applied to assess the association between ALG3 and immune infiltration. CCK8, MTT, and transwell assays were used to investigate the role of ALG3 downregulation in HCC cell lines.

**Results:** qRT-PCR, WB, and IHC proved ALG3 was highly overexpressed in HCC tissues. The Kaplan–Meier analysis verified the overexpression of ALG3 was related to poor overall survival (*p* < 0.001). Multivariate cox regression analysis showed that the high ALG3 expression was an independent risk prognostic factor. GSEA and TIMER2.0 predicted that ALG3 participates in cell differentiation and cycle and correlates with immune cell infiltration. Transwell assay results showed that ALG3 silencing also impaired the invasion ability of HCC cells.

**Conclusion:** ALG3 was overexpressed and considered a potential indicator of survival in HCC, and our findings provided a novel therapeutic target for HCC.

## Introduction

Liver cancer is the second most common cause of cancer-related mortality (Bray et al., 2018), and the highest incidence rates are found in Asia and Africa (McGlynn et al., 2021). As one of the top five deadliest cancers in the world, the incidence of liver cancer has increased annually and is an increasingly frequent cause of cancer death (Siegel et al., 20192019). Hepatocellular carcinoma (HCC) is the primary type of liver cancer, accounting for about 75% of the total cancer deaths (Petrick et al., 2020). At an early stage, surgical resection and organ transplantation remain the only curative options for HCC, but the 5-year recurrence rate is around 50% (Pinato et al., 2017) due to the high proportion of metastasis (Lu et al., 2019). The lack of survival benefits from conventional drugs and sorafenib suggests the urgent need to find new biological therapeutic strategies (Faivre et al., 2020).

Following the successful application of immune checkpoint blockers (ICBs) in several tumor types, lots of studies are researching ICBs alone or in combination with other treatments in patients with HCC (Pinter et al., 2021). Currently, another four targeted therapies have been approved for HCC, except for sorafenib: lenvatinib (Zhao et al., 2020), regorafenib (Grothey et al., 2020), cabozantinib (Shang et al., 2021), and ramucirumab (Zhu et al., 2019), but immunotherapy is only effective in a subset of HCC patients (Ruf et al., 2021). Hence, a deeper understanding of the molecular mechanisms and biological pathways involved in the development of HCC will be conducive to exploring new biomarkers and improving survival time in HCC patients.

Glycosylation is involved in cell–matrix interaction, recognition, signal recognition, and synaptic signaling and plays an important role in many cellular processes (Varki, 2017). Targeting glycosylation is considered as a new way of cancer drug discovery (Costa et al., 2020). The different expressions of glycosyltransferases in several cancer types have been verified as a potential indicator of survival and treatment targets (Song et al., 2020). Alpha-1,3-mannosyltransferase (ALG3) encodes an endoplasmic reticulum-localized ALG3 family member that participates in N-glycan synthesis (Bloch et al., 2020). Researchers have observed that ALG3 contributes to high mannose-type N-glycans in several cancers, and mannose-type N-glycan upregulation has been shown to be related to cancer progression (Silva et al., 2020). Studies have demonstrated that ALG3 is overexpressed in oral squamous cell carcinoma (Shao et al., 2021), non–small cell lung cancer (Ke et al., 2020), and breast cancer (Yang et al., 2018; Sun et al., 2021) and contributes to drug resistance in the acute myeloid leukemia (Liu et al., 2020a). However, the expression of ALG3 in HCC is unknown, and its prognosis values and correlations with clinicopathologic features of HCC patients need to be further explored.

In our study, public databases were first utilized to identify the mRNA expression of ALG3 and its relation to the prognosis. Then, clinical cases, tissues, and cell lines were applied to prove the ALG3 expression and the relationship between ALG3 and clinicopathologic characteristics. Functional enrichment analysis and TIMER2.0 were utilized to explore the potential mechanisms and their relations with immune infiltrations.

## Materials and Methods

### Cell Culture

The human liver cell line LO2 and hepatocellular cell lines SNU398, SNU449, MHCC97H, Hep3B, PLC/PRF/5, and HepG2 were purchased from the ATCC (Manassas, VA, United States). PLC/PRF/5, Hep3B, MHCC97H, and HepG2 cell lines were cultured in complete Dulbecco’s modified Eagle’s medium (Gibco, United States), and SNU398 and SNU449 cells were cultured in RPMI1640 medium (Gibco, United States); all cells were supplemented with 10% fetal bovine serum (FBS) at 37°C in a humidified incubator with 5% CO_2_ in the air.

HCC cell lines (Hep3B, HepG2, and SNU398) were transfected with siRNAs targeting ALG3 or control siRNA (HIPPOBIO, China) using Lipofectamine 2000 Reagent (Invitrogen, United States). The efficiency of the knockdown was confirmed using qRT-PCR analysis. The siRNA used were as follows: si-NC: 5′-UUC​UUC​GAA​GGU​GUC​ACG​UTT-3′, ALG3 siRNA#1: 5′-GGU​UUC​GUG​UAC​AUC​UUU​AUG-3′, and ALG3 siRNA#2: 5′-GGA​CCU​GAG​UCU​ACC​CUC​AGG-3’.

### Patients and Specimens

Eight fresh tissue specimens of HCC and 115 paraffin-embedded specimens, which were pathologically diagnosed with HCC and obtained from Guangdong Provincial People’s Hospital (GDPH) (Guangdong, China), were enrolled in this study. The intrahepatic recurrence and extrahepatic metastasis were both considered as recurrences. The use of these clinical materials for our study was approved by the Research Ethics Committee of (GDPH) and by each patient. The clinicopathologic features are summarized in Supplementary Table S1.

### Tumor IMmune Estimation Resource Database

The online database “Tumor IMmune Estimation Resource” (TIMER2.0; https://cistrome.shinyapps.io/timer/) was used to assess the expression levels of ALG3 mRNA in different cancer patients and the relationship between the ALG3 expression and immune cells in HCC (Li et al., 2020a).

### Human Protein Atlas Database

The HPA (https://www.proteinatlas.org/) is a public server, including patients' proteomic and RNA-seq data in every single-cell, organ, and tissue from human normal or abnormal tissues. Thus, ALG3 was analyzed in single-cell types and single-cell lines by using the search term “ALG3” (Thul and Lindskog, 2018).

### Data Acquisition

Gene expression data were acquired from The Cancer Genome Atlas (TCGA) database up to May 2021 (https://portal.gdc.cancer.gov). The GSE14520 and GSE124535 datasets were downloaded from the Gene Expression Omnibus (GEO) database (https://ncbi.nlm.nih.gov/geo). The Supplementary Table S3 dataset was obtained from HCCDB (Lian et al., 2018) for expression validation.

### RNA Extraction, Reverse Transcription, and Quantitative Real-Time PCR

All RNA samples from the HCC cell lines and clinical patient’s tissues were extracted using the TRIzol reagent (Invitrogen, Carlsbad, CA, United States). For the procedure, 1 µg RNA was used for cDNA synthesis primed with random hexamers. cDNAs were amplified by SYBR-Green in a CFX96 real-time system C1000 cycler. Results of amplification were downloaded and analyzed with EXCEL, and GAPDH was used for internal control. Each sample was tested in triplicate at least three times. All fold changes were calculated through relative quantification 2^[(GAPDHCq)—(ALG3Cq)]. The detailed primers sequences are provided in Supplementary Table S2.

### Western Blot

Tissue sample and cell lines (LO2, SNU449, SNU398, PLC/PRF/5, MHC97-H, Hep3B, and HepG2) of total protein were obtained in lysis buffer. Protein density was measured using the BCA test kit (Fdbio Science, China), according to the manufacturer’s suggested protocols. Equal amounts (40 µg) of protein were loaded to SDS–PAGE and transferred to the NC membrane. After being blocked with 5% milk for 45 min, the membranes were incubated overnight at 4° with antibodies against ALG3 (1:1,000 dilution; NO. 20290–1-AP, Proteintech, Manchester, United Kingdom) and GAPDH (1:1,000 dilution; CST, Shanghai, China); next day, the membranes were washed five times for 5 min with TBST (2% Tween) and then incubated with anti-rabbit IgG for 45 min at room temperature. The expression of the target protein was detected by chemiluminescence.

### Immunohistochemistry Analysis

IHC staining was applied on 115 paraffin sections of clinical HCC specimens using the ALG3 antibody (Sigma, HPA045130, 1:200). Two pathologists independently assessed the specimens, who were blinded to the details, on the basis of staining degree and the proportion of positive tumor cells. The percentage of positive cells was scored as follows: 0: <5%; 1: 5%–25%; 2: 5%–50%; 3:50%–75%; and 4: >75%. The immunostaining intensity grade was as follows: 0 (negative), 1 (weak), 2(moderate), and 3(strong). The staining degree was multiplied by the percentage of positive cells as the staining score. In this study, an ALG3 score ≥8 was defined as a high expression.

### Functional and Pathway Enrichment Analysis

The STRING (https://string-db.org/) database was applied to construct a protein–protein interaction (PPI) network for ALG3. R language was used to visualize the Gene Ontology (GO) and Kyoto Encyclopedia of Genes and Genomes (KEGG) pathway. *p* < 0.05 was considered significant.

### Gene Set Enrichment Analysis

Gene Set Enrichment Analysis (GSEA) (http://www.gsea-msigdb.org/gsea/index.jsp) was used to evaluate the potential biological mechanisms. *p* < 0.05 and FDR<0.25 were the criteria to determine a significant differential expression.

### Cell Proliferation Assay

The cellular proliferation was evaluated by the CCK-8 (Beyotime, China) and MTT (Sigma-Aldrich, United States) assays, according to the manufacturer’s instructions. Briefly, the cells successfully transfected with ALG3 siRNA or control siRNA were seeded in 96-well plates, with three replicate wells in each group. The optical density (OD) values of the cells were measured every 24 h. Before the measurement, 10 µL of CCK8 or MTT (5 mg/ml) solution was added to the medium and incubated for 2 h. The OD values were measured under the absorbance of 450 or 490 nm by using a microplate reader (Bio-Rad Laboratories, Hercules, CA, United States). Finally, a growth curve was made by GraphPad Prism 8.

### Cell Invasion Assay

Transwell chambers (8 µM, Corning Costar, China) were used to detect the invasion abilities of Hep3B cells. The upper chamber was pre-coated with Matrigel to form a matrix barrier, and 2 × 10^4^ cells suspended in 200 µl serum-free DMEM medium were seeded in triplicate into the upper chamber of 24-well chambers. The under compartment included a 600 µl DMEM medium with 10% FBS. After cultivation in a 37°C incubator for 24 h, the chambers were rinsed with PBS gently to remove the cells glued to the upper surface. After being fixed and stained, the cells were photographed in three fields randomly under a microscope.

### Statistical Analysis

SPSS version 25.0 and GraphPad Prism 8 (San Diego, CA, United States) were used for statistical analyses. Student’s test, Kaplan–Meier survival curves were plotted by GraphPad Prism 8.0.1 to analyze the difference between the two groups. Cox regression analysis was performed to identify the independent prognostic factors of patients with hepatocellular carcinoma. *p* < 0.05 indicates a significant difference.

## Results

### Elevated Expression of Alpha-1,3-Mannosyltransferase in Variety of Malignant Tumors

**FIGURE 1:**
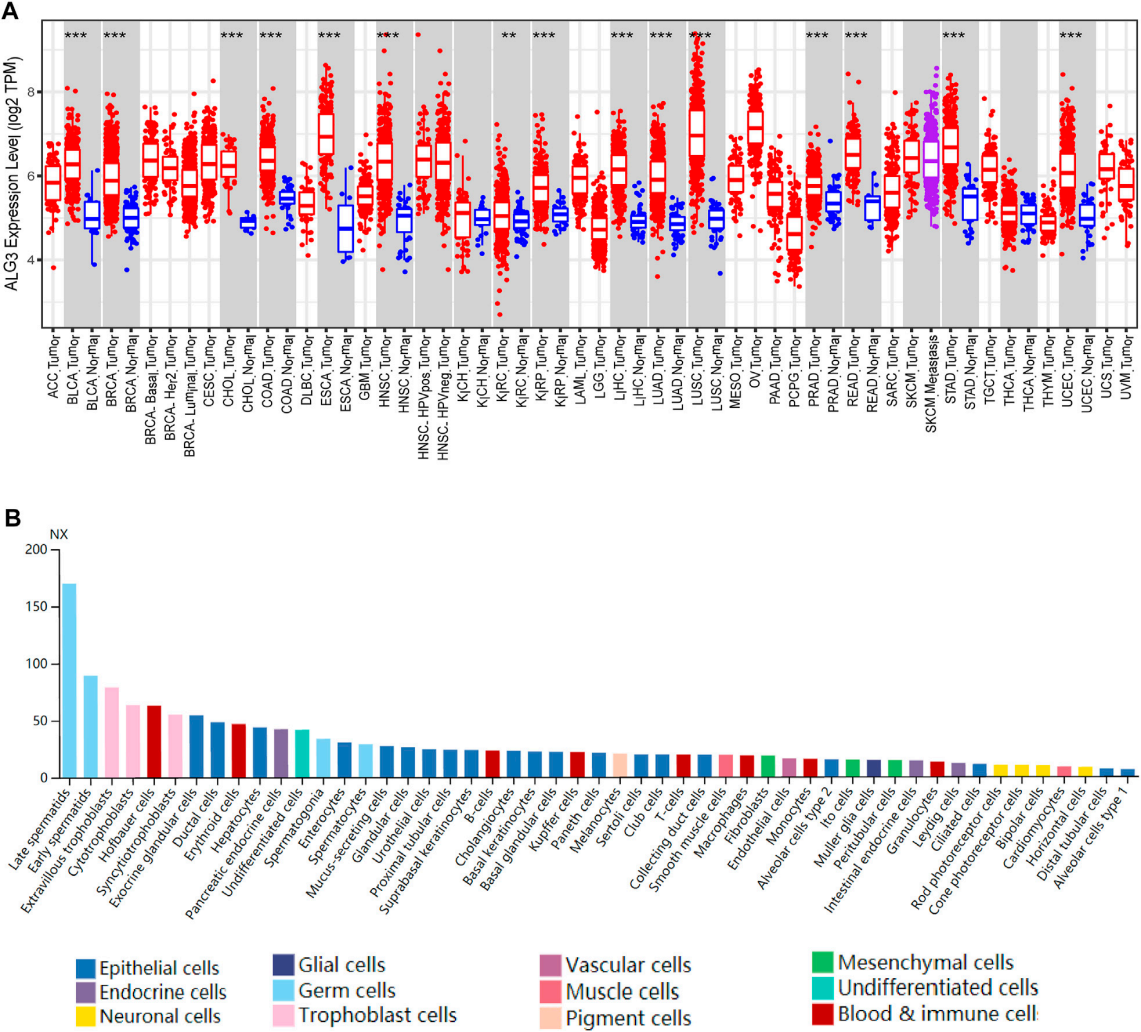
Expression of ALG3 in different cancer types. **(A)** ALG3 mRNA expression levels across different cancer types in the TIMER database. **(B)** ALG3 expression in single cell type lines analyzed by HPA. (**p < 0.01 and ***p < 0.001) ACC, adrenocortical carcinoma; BLCA, bladder urothelial carcinoma; BRCA, breast invasive carcinoma; CESC, cervical squamous cell carcinoma; CHOL, cholangiocarcinoma; COAD, colon adenocarcinoma; DLBCL, lymphoid neoplasm diffuse large B-cell lymphoma; ESCA, esophageal carcinoma; GBM, glioblastoma multiforme; HNSC, head and neck squamous cell carcinoma; KICH, kidney chromophobe carcinoma; KIRC, clear cell renal cell carcinoma; KIRP, kidney renal papillary cell carcinoma; LAML, acute myeloid leukemia; LGG, brain lower grade glioma; LIHC, liver hepatocellular carcinoma; LUAD, lung adenocarcinoma; LUSC, lung squamous cell carcinoma; MESO, mesothelioma; OV, ovarian serous cystadenocarcinoma; PAAD, pancreatic adenocarcinoma; PRAD, prostate adenocarcinoma; PCPG, pheochromocytoma and paraganglioma; READ, rectal adenocarcinoma; SARC, sarcoma; SKCM, skin cutaneous melanoma; STAD, stomach adenocarcinoma; TGCT, testicular germ cell tumor; THCA, thyroid carcinoma; THYM, thymoma; UCEC, uterine endometrial carcinoma; UCS, uterine carcinosarcoma; UVM, uveal melanoma.

We chose TIMER2.0 and HPA cancer databases to investigate the expression of ALG3 mRNA. As shown in Figure 1A, our result showed that ALG3 was significantly expressed in multiple cancer types compared with normal samples, including BLCA (*p* = 8.82E-12), BRCA (*p* = 2.72E-55), CHOL (*p* = 4.51E-09), COAD (*p* = 4.29E-23), ESCA (*p* = 6.73E-08), HNSC (*p* = 5.54E-25), KIRC (*p* = 0.007), KIRP (*p* = 1.95E-13), LIHC (*p* = 1.55E-26), LUAD (*p* = 8.58E-32), LUSC (*p* = 1.02E-30), PRAD (*p* = 7.86E-12), READ (*p* = 5.17E-07), STAD (*p* = 2.14E-19), and UCEC (*p* = 2.29E-19). In the HPA public database, the mRNA expression of ALG3 was upregulated in germ cells, trophoblast cells, blood and immune cells, and epithelial cells. Importantly, ALG3 was highly expressed in hepatocytes, as shown in Figure 1B. In short, our results suggested that ALG3 was overexpressed in different cancers including liver hepatocellular carcinoma tissues and liver cell lines.

### Alpha-1,3-Mannosyltransferase Is Markedly Upregulated in Hepatocellular Carcinoma

**FIGURE 2:**
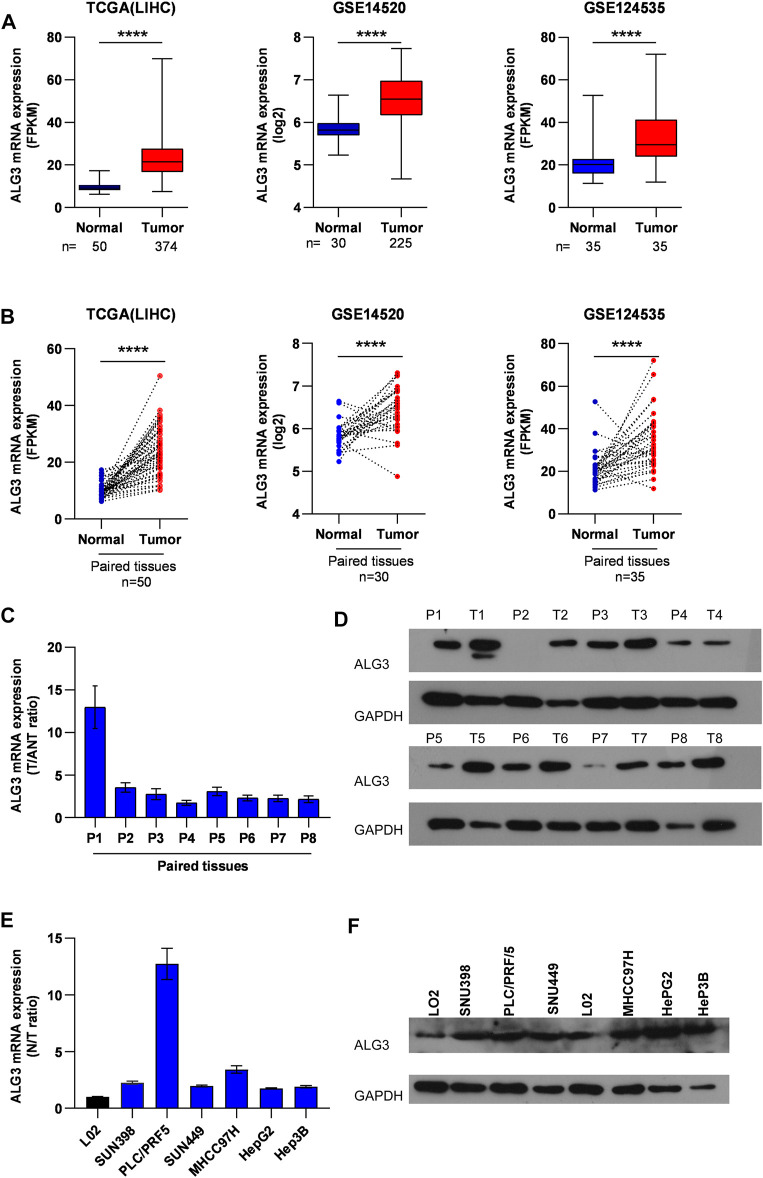
Upregulation of ALG3 in HCC. **(A)** ALG3 mRNA expression levels were markedly overexpressed in unpaired HCC tissues, as indicated by the TCGA LIHC dataset and GEO HCC datasets GSE14520 and GSE124535. **(B)** ALG3 mRNA was upregulated in paired HCC tissues and adjacent normal tissues in the TCGA LIHC dataset and GSE14520 and GSE142535 datasets. RT-PCR **(C,E)** and WB **(D,F)** analysis of ALG3 expression in paired HCC tissues and non-tumor adjacent tissues and in the liver cancer cell line. GAPDH was used as a loading control. (**p < 0.01, ***p < 0.001, and ****p < 0.0001)

To research the role of ALG3 in hepatocellular carcinoma, first, we explored the ALG3 expression on online public human hepatocellular carcinoma datasets of the GEO and TCGA databases. Obviously, the result revealed that compared with normal controls, ALG3 was indeed upregulated in HCC tissues and in paired HCC tissues (Figures 2A,B). Other data sets from HCDDB also demonstrated the differential expression of ALG3 in HCC (Supplementary Table S3). To further confirm both mRNA and protein expression levels of the ALG3 expression in HCC, qRT-PCR and Western blot were conducted for the specimens from our hospital. The results showed that ALG3 was significantly overexpressed in HCC tissues compared with non-tumor adjacent tissues (Figures 2C,D). In addition, as shown in Figures 2E,F, the differential expression of ALG3 has been further verified in liver cell lines. Therefore, these results indicate that the ALG3 expression greatly increases in HCC.

### Relationships Between Alpha-1,3-Mannosyltransferase and Clinicopathologic Features of Hepatocellular Carcinoma Patients

**FIGURE 3:**
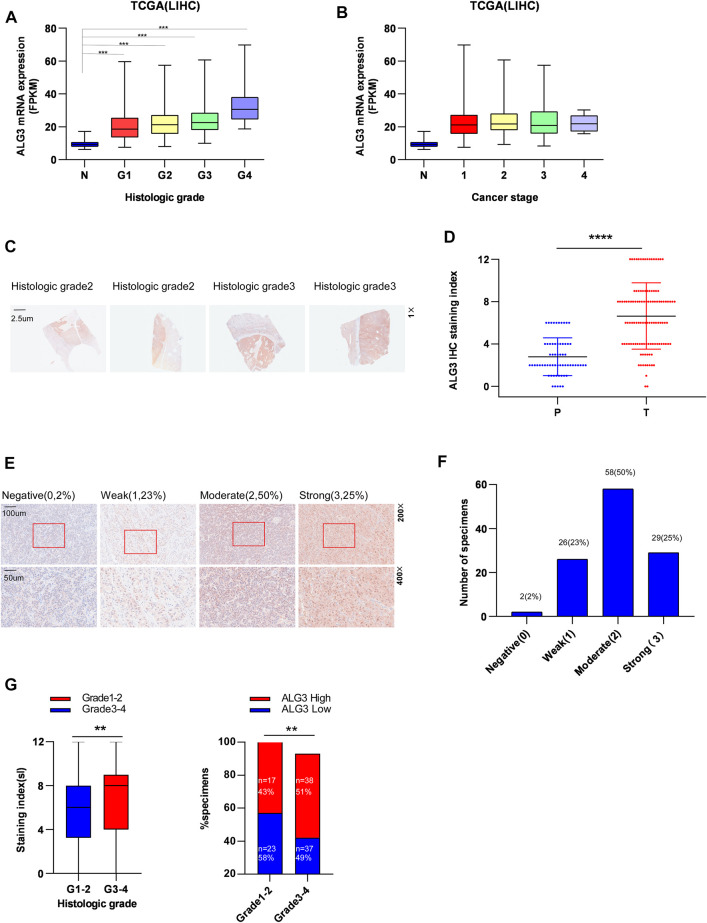
Relationship between ALG3 and clinicopathologic features of HCC patients. The expression of ALG3 is ordered by histologic grade **(A)** and cancer stage **(B)** in TCGA. **(C)** Representative images of ALG3 staining in paired HCC tissues. **(D)** Detailed IHC staining index. **(E)** IHC analysis of ALG3 protein expression in HCC tissues of different representative images. The bottom shows a double magnification of part of the aforementioned image. **(F)** Number of cases with different staining intensities. **(G)** ALG3 expression levels in different histologic grade specimens. (**p < 0.01, ***p < 0.001, and ****p < 0.0001).

We performed a subgroup analysis of TCGA data based on clinicopathologic characteristics. Importantly, the ALG3 expression based on tumor grade and cancer stage performed significant differences (Figures 3A,B). To better understand how ALG3 protein affected the prognosis of HCC patients, we further performed IHC staining in 115 paraffins obtained from GDPH. The baseline clinicopathologic information of 155 HCC patients is showed in Supplementary Table S1, and the association of the ALG3 expression with other pathological features is showed in Table 1. As shown in Figures 3C,D, the tumor and non-tumor tissues were notably in different degrees of staining, and ALG3 staining was stronger in advanced-grade versus early-grade carcinoma. The representative images of different ALG3 protein expressions are shown in Figure 3E. The detailed case information with all staining intensities is displayed in Figures 3D,F. IHC results revealed that ALG3 protein was mainly localized in the cytoplasm. Subgroup analysis showed that the ALG3 expression was higher in histologic grades III- IV than in histologic grades I and II (Figure 3G) and was obviously associated with the histologic grade (*p* = 0.0097).

**TABLE 1:** Association between the ALG3 expression and other clinicopathologic features.

Feature of HCC	No. of patients (%)	ALG3 expression		*p-*values
		Low expression (60)	High expression (55)	
Age (years)				0.047#
≤60	60	26	34	
>60	55	34	21	
Gender				0.930#
Male	79	41	38	
Female	36	19	17	
Histology classification				0.404#
I–II	40	23	17	
III–IV	75	37	38	
TNM stage (AJCC)				0.112#
T1–T2	89	50	39	
T3–T4	26	10	16	
Vital status				0.002#
Alive	64	25	39	
Dead	51	35	16	
Recurrent status				0.473#
Yes	79	43	36	
No	36	17	19	
Tumor size (cm)				0.827#
<5	43	23	20	
≥5	72	37	35	
Tumor number				0.181#
Single	90	44	46	
Multiple	25	16	9	
Tumor capsular				0.585#
Complete	96	49	47	
Incomplete	19	11	8	
Cut edge				1*
Negative	107	56	51	
Positive	8	4	4	
Vascular tumor emboli				0.343#
Present	45	21	24	
Absent	70	39	31	
Serum AFP (ng/ml)				0.797#
<400	76	39	37	
≥400	39	21	18	
HBV DNA				0.473#
<500	79	43	36	
≥500	36	17	19	
HBsAg				0.160#
Negative	41	25	16	
Positive	74	35	39	
Postoperative complication				0.335*
No	102	51	51	
Postoperative hemorrhage	4	3	1	
Bile leakage	3	3	0	
Liver failure	1	0	1	
Others	5	3	2	
Postoperative adjuvant therapy				0.582*
HIPEC	8	6	2	
TACE	39	19	20	
Sorafenib	3	2	1	
No	65	33	32	

TBX3 immunohistochemical score ≥8 was regarded as high expression.

#*p* values and *p values were calculated with the chi-squared test and Fisher’s exact test, respectively.

### Overexpression of Alpha-1,3-Mannosyltransferase Is Correlated With Poor Prognosis in Hepatocellular Carcinoma

**FIGURE 4:**
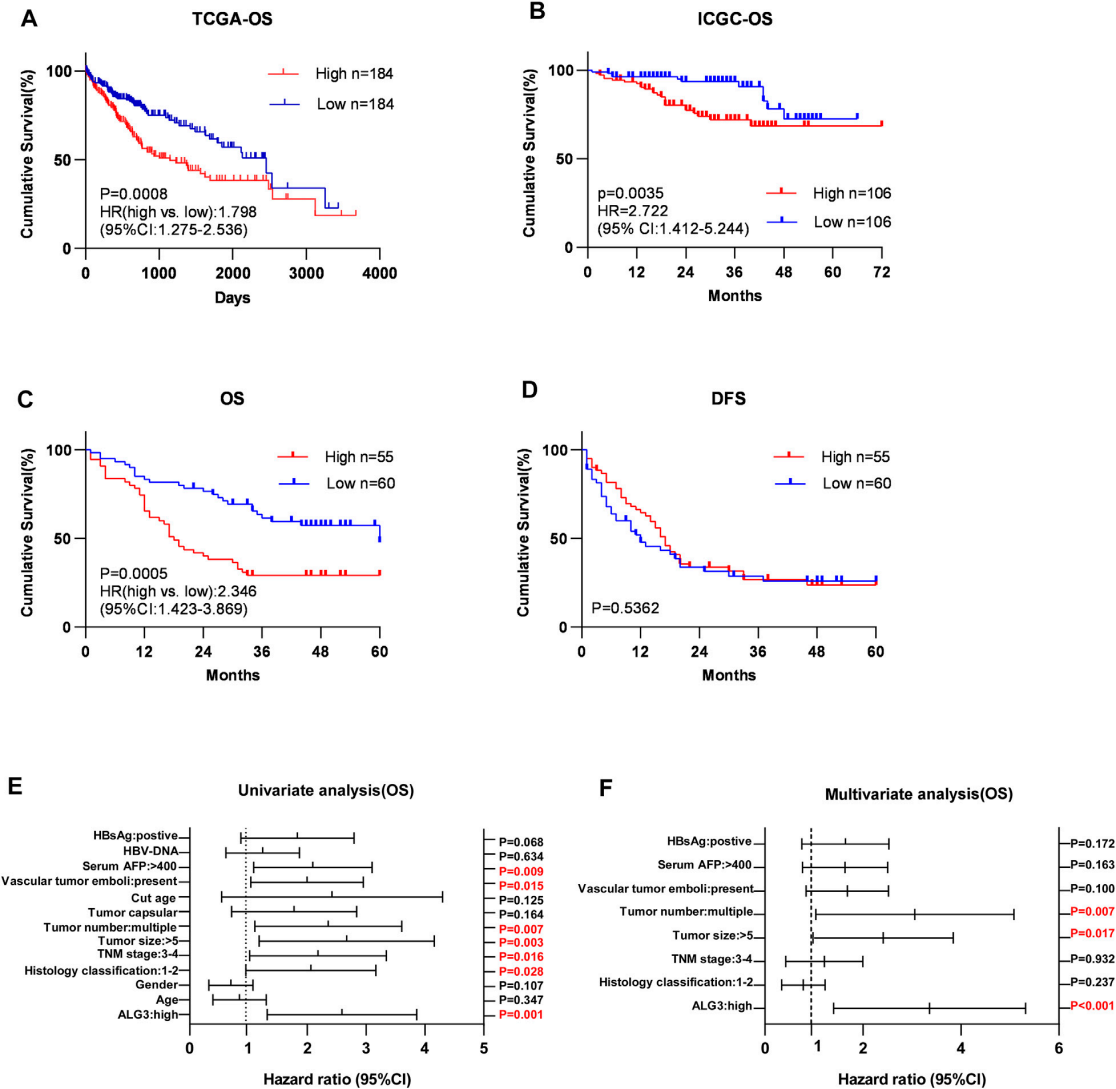
Over-expression of ALG3 is correlated with poor prognosis in HCC. Kaplan–Meier analysis of 5-year overall survival for HCC in the TCGA-LIHC dataset **(A)** and ICGC dataset **(B)**. Kaplan–Meier analysis of OS **(C)** and DFS **(D)** for GHPD stratified by low and high ALG3 expressions (*n* = 115, log-rank test). Univariate**(E)** and multivariate**(F)** Cox regression analyses to assess the significance of the association between ALG3 and 5-year overall survival in the presence of other clinical variables.

Kaplan–Meier survival curves for HCC in TCGA, LIHC, and ICGC datasets indicated that the overall survival of HCC patients with a low expression of ALG3 was longer than that of high expression (Figures 4A,B ). According to IHC staining results and the grouping criteria, the specimens were divided into high-expression (55 patients) and low-expression groups (60 patients). Patients with high ALG3 expression had shorter overall survival (*p* = 0.0005; hazard ratio (95% CI) = 2.346 (1.423–3.869) but had the same disease-free survival than the low ALG3 expression group (*p* = 0.5362) (Figures 4C,D). Furthermore, univariate and multivariate analyses were applied to estimate the influence of ALG3 and pathological characteristics on HCC patients (Table 2). Univariate analysis showed that ALG3 expression, histology classification, TNM stage (AJCC), tumor size, tumor number, vascular tumor emboli, serum AFP, and HBsAg were the potential independent prognostic factors for HCC patients (Figure 4E). Multivariate analysis indicated that ALG3 expression, tumor number, and tumor size were the independent factors of 5-year overall survival (Figure 4F). Collectively, these findings indicated that a higher expression of ALG3 might promote HCC progression, leading to poor clinical prognosis.

**TABLE 2:** Univariate and multivariate analysis of overall survival between the ALG3 expression and other clinicopathologic features.

Feature	No.	Univariate analysis (OS)			Multivariate analysis (OS)		
		EXP(B)	95% CI	*p*	EXP(B)	95% CI	*p*
ALG3		2.380	1.434–3.949	0.001	2.905	1.669–5.508	<0.001
High expression	55						
Low expression	60						
Age (years)		0.766	0.439–1.336	0.347			
≤50	29						
>50	86						
Gender		0.633	0.363–1.103	0.107			
Male	79						
Female	36						
Histology classification		1.863	1.068–3.250	0.028	0.701	0.389–1.263	0.237
I–II	40						
III–IV	75						
TNM stage		1.975	1.137–3.431	0.016	1.031	0.531–2.072	0.932
T1–T2	89						
T3–T4	26						
Tumor size (cm)		2.394	1.339–4.278	0.003	2.129	1.143–3.965	0.017
<5	43						
≥5	72						
Tumor number		2.136	1.233–3.701	0.007	2.614	1.297–5.270	0.007
Single	90						
Multiple	25						
Tumor capsular		1.562	0.833–2.930	0.164			
Complete	96						
Incomplete	19						
Cut edge		1.934	0.832–4.496	0.125			
Negative	107						
Positive	8						
Vascular tumor emboli		1.844	1.126–3.021	0.015	1.542	0.920–2.582	0.100
Present	45						
Absent	70						
Serum AFP (ng/ml)		1.932	1.177–3.171	0.009	1.482	0.853–2.573	0.163
<400	76						
≥400	39						
HBV DNA		1.135	0.673–1.913	0.634			
<500	79						
≥500	36						
HBsAg		1.662	0.964–2.867	0.068	1.481	0.843–2.602	0.172
Negative	41						
Positive	75						

### Functional and Pathway Enrichment Analyses of Alpha-1,3-Mannosyltransferase

**FIGURE 5:**
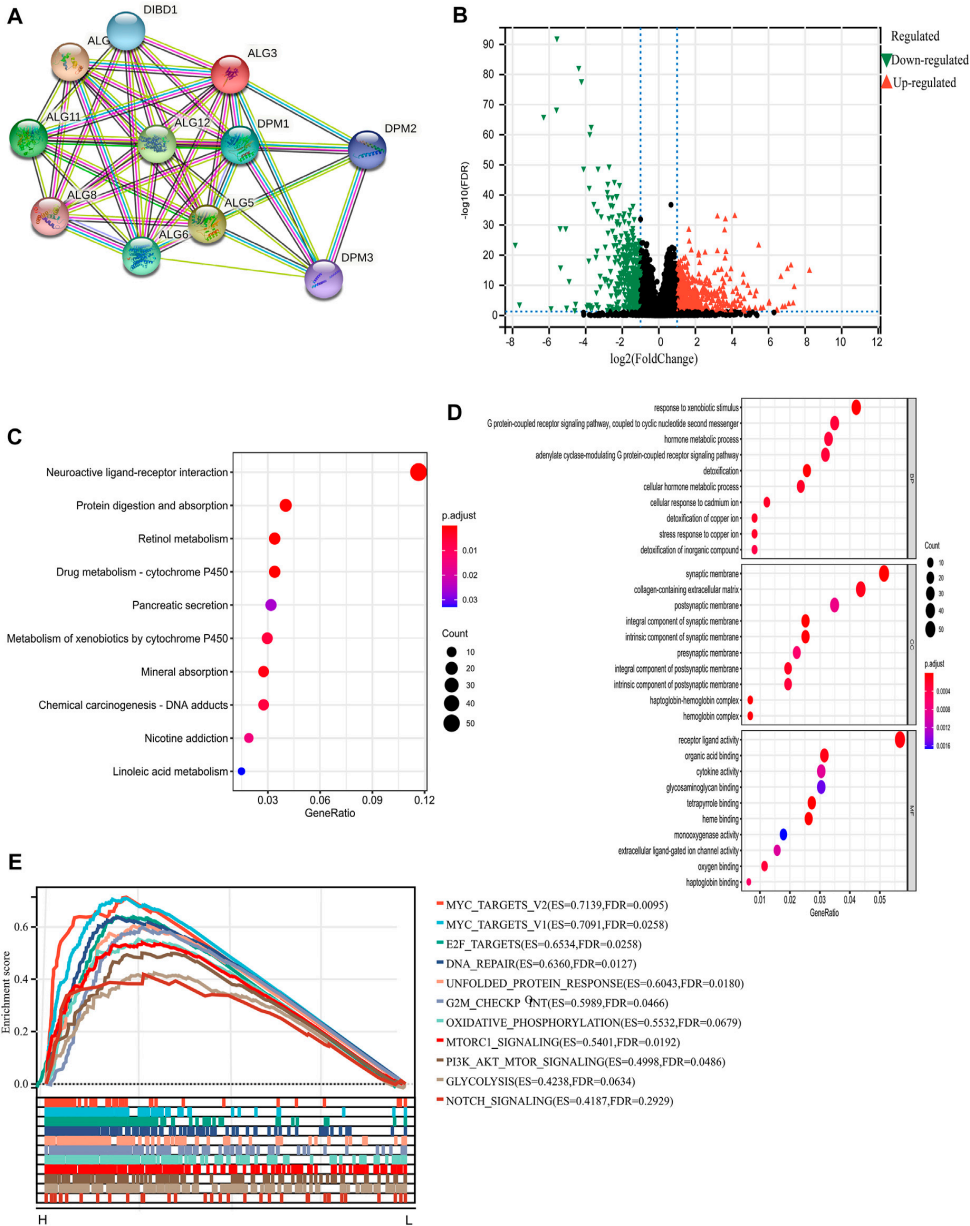
PPI and functional enrichment analyses of ALG3 in HCC. **(A)** STRING tool to construct a network of the interaction between ALG3 and other proteins. **(B)** Volcano plot depicting the DEGs. **(C)** Kyoto Encyclopedia of Genes and Genomes (KEGG) pathway, **(D)** Gene Ontology (GO) enrichment, and **(E)** gene set enrichment analysis (GSEA) of ALG3.

We intend to figure out the potential mechanism on which ALG3 promotes the progression of HCC. First, we used STRING tools to construct a PPI network to identify genes associated with ALG3 and found that ALG3 was connected with DIBD1, DPM1-3, ALG5, ALG6, ALG8, ALG9, ALG11, and ALG12 (Figure 5A). Next, the volcano plot depicted the analysis of the differentially expressed proteins between low and high ALG3 expression groups (Figure 5B). KEGG and GO analysis were utilized to explore the potential biological mechanism of ALG3. Based on GO biological process analysis, the DEGs were significantly enriched in response to the xenobiotic stimulus, G protein-coupled receptor signaling pathway, hormone metabolic process, adenylate cyclase-modulating G protein-coupled receptor signaling pathway, collagen-containing extracellular matrix, synaptic membrane, and receptor ligand activity (Figure 5D).

KEGG pathway analysis results indicated that these DEGs were mainly enriched in neuroactive ligand-receptor interaction, protein digestion and absorption, retinol metabolism, and drug metabolism-cytochrome P450 (Figure 5C). In addition, we chose the “hallmark gene set” for GSEA based on the expression of ALG3 to perform the different enriched pathways between upregulated and downregulated ALG3 expressions in HCC samples. The positively enriched biological pathways include MYC targets, E2F targets, DNA repair, G2M checkpoint, and MTORC1 signaling (Figure 5E).

### Relationship Between Alpha-1,3-Mannosyltransferase Expression and Immune Infiltration in Hepatocellular Carcinoma

**FIGURE 6:**
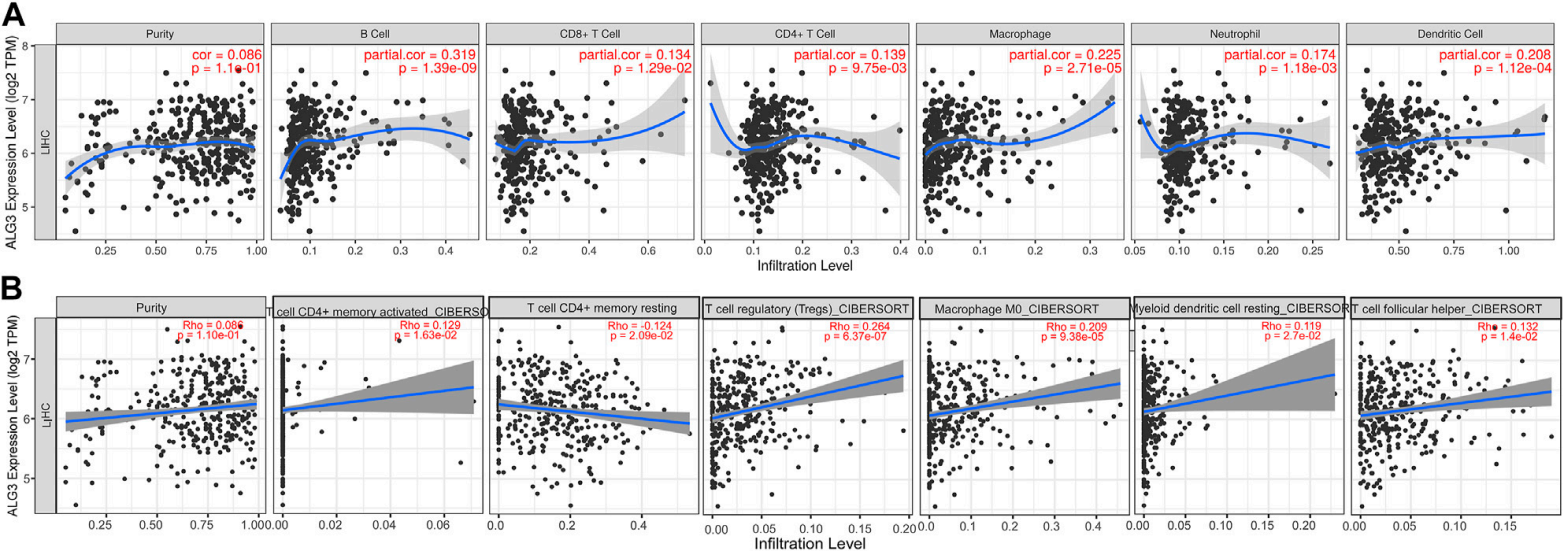
Correlations of ALG3 expression with TILs in HCC. Correlations between the ALG3 expression and TILs were verified with TIMER web server **(A)** and CIBERSORT **(B)**.

As we all know, immune infiltration has a huge impact on the occurrence and development of tumors (Sun et al., 2020). To better understand the roles of ALG3 in immune infiltration, the TIMER web server was used to evaluate the relationships between ALG3 and the immune cells of HCC, including dendritic cells. As shown in Figure 6A, the high ALG3 expression was significantly related to the infiltration of neutrophils (R = 0.174 and *p* = 1.18e-03), B cells (R = 0.319 and *p* = 1.39e-09), dendritic cells (R = 0.208 and *p* = 1.12e-04), CD8^+^ T cells (R = 0.134 and *p* = 1.29e-02), macrophages (R = 0.225 and *p* = 2.71e-05), and CD4^+^ T cells (R = 0.139 and *p* = 9.75e-03). CIBERSORT’s data also validated the high expression of ALG3 was mainly correlated with T-cell subsets: CD4^+^ T-cell memory activated and rest, Tregs, and T helper cells (Figure 6B). However, more detailed experimental research is needed to prove this conclusion.

### Knockdown of Alpha-1,3-Mannosyltransferase Impeded the Invasion of Hepatocellular Carcinoma Cells

**FIGURE 7:**
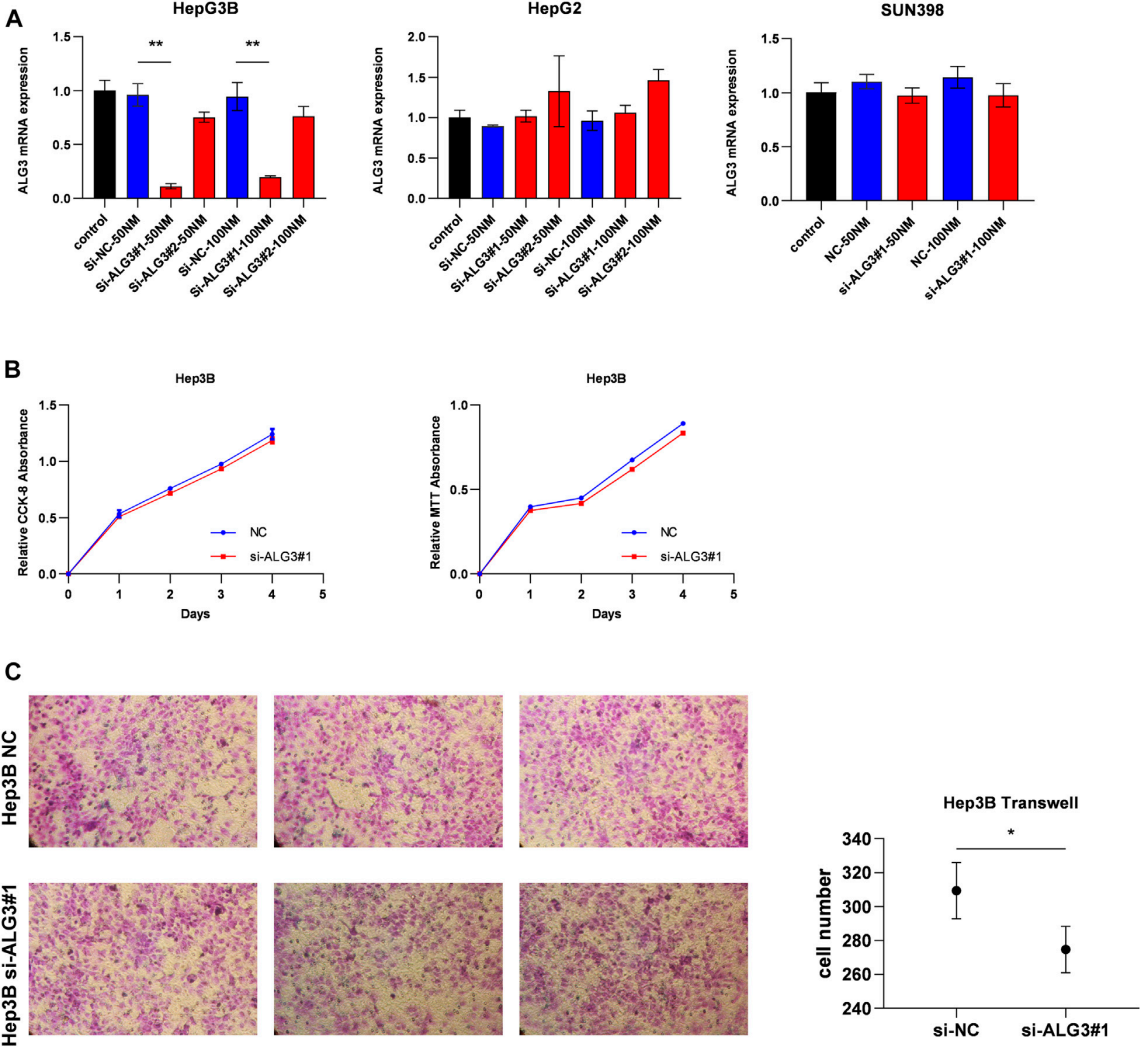
ALG3 promotes HCC cell invasion. **(A)** Quantitation of the levels of ALG3 mRNA in HepG2, Hep3B, and SNU398 cells by qRT-PCR after transfection for 48 h with different siRNAs targeting ALG3 (si#1 and si#2) or a control siRNA. **(B)** CCK-8 and MTT assays evaluated the proliferation of Hep3B cells after the knockdown of ALG3. **(C)** Invasion number of Hep3B cells was decreased with ALG3 down-regulation. (*p < 0.05 and **p < 0.01)

First, we verified the mRNA and protein expression of ALG3 in HCC cell lines. According to Figures 2E,F, we have selected HepG2, Hep3B, and SNU398 to construct transient cell lines for the function test. As shown in Figure 7A, we verified that ALG3 was successfully silenced by siRNA#1 in Hep3B cells (*p* < 0.01). Next, we used the CCK-8 and MTT assays to explore the effects of ALG3 silencing on the proliferation of Hep3B cells. Our results showed that siRNA-induced silencing did not significantly inhibit the proliferation of Hep3B cells (Figure 7B), but the overall trend is slightly downward. In addition, transwell assay results showed that ALG3 silencing impaired the invasion and migratory ability of Hep3B cells (Figure 7C). These results suggested that high expression of ALG3 was associated with the proliferation and invasion of HCC cell lines.

## Discussion

Immunotherapy and chemotherapy have a huge impact on hepatocellular carcinoma therapy with the development of technology (Kennedy and Salama, 2020). Currently, studies have shown that the anti-PD-1 antibody combined with locoregional treatments or other molecular-targeted agents is an effective therapy for HCC (Xu et al., 2018), but the precise efficacy and benefit populations have yet to be assessed. Additional reliable novel therapeutic targets need to be found. Previous studies have shown that ALG3 is a high correlation with congenital disorders of glycosylation (Marques-da-Silva et al., 2017; Bian et al., 2020; Ferrer et al., 2020; Alsharhan et al., 2021). In recent years, within the tumor cells, different expressions of glycosyltransferases have been proved as promising biomarkers and potential treatment targets, and glycosylated proteins have attracted increasing attention from researchers. Currently, alpha-fetoprotein (AFP) for liver cancer (Liu et al., 2017), prostate-specific antigen (PSA) for prostate cancer (Wilt and Dahm, 2015), and carcinoembryonic antigen (CEA) for colon cancer (Konishi et al., 2018) are all glycosylated proteins that have been used as biomarkers in clinical application. According to previous studies, ALG3 promotes the invasion, migration, and proliferation of oral squamous cell carcinoma (Shao et al., 2021), non-small cell lung cancer (Ke et al., 2020), and breast cancer (Yang et al., 2018; Sun et al., 2021), but the expression of ALG3 and its relation with the prognosis of HCC patients remains unknown.

First, we discovered that ALG3 was highly expressed in several malignant tumors, and high expressions can affect the prognosis of patients. In order to find out the role of ALG3 in HCC, we used the TCGA, GEO, and HCDDB datasets to investigate its expression in HCC patients and accordingly found that ALG3 was overexpressed in HCC. Then, we verified the mRNA and protein expression in eight fresh tissue specimens and L02, SNU398, SNU449, PLC/PRF/5, MHCC-97H, HepG2, and Hep3B cell lines. We further proved that both mRNA and protein were higher than in the control group. Next, we conducted the IHC analysis in HCC specimens’ slices. IHC results showed the abnormal expression of ALG3 was positively correlated with the advanced histologic grade and cancer stage. Moreover, Kaplan–Meier analyses proved higher expression of ALG3 led to the shorter OS, and multivariate cox regression analysis identified the ALG3 as an independent prognostic factor for HCC patients. To sum up, our findings showed that ALG3 is upregulated in HCC patients and could be regarded as a potential prognosis biomarker.

Then, we constructed PPI networks for ALG3 to search for the relevant genes which play a great role in HCC progression. DPM1, one of the relevant genes, is a vital sensor molecule related to the regulation of cytosolic Ca^2+^ levels in the maintenance of B-cell functions and a potential prognostic tumor marker of HCC (Li et al., 2020b). ALG5 was reported as a prognostic biomarker for early tumor progression in advanced high-grade serous ovarian cancer (HGSOC) ALG5 (Lu et al., 2021). A previous study indicated that ALG9 was regulated by lncRNA MEG3 and participated in the drug resistance mechanism in acute myeloid leukemia (Yu et al., 2020). Moreover, ALG11 was confirmed as the independent risk factor for the prognosis of HCC in Liu et al. (2021). These results indicated that co-expressed genes played an important role in various cancers, but the synergistic pathways of ALG3 and its correlated genes in HCC remained elusive. Next, we used GO and KEGG to analyze the pathways which were affected by DEGs. The result showed that ALG3 is mainly related to the neuroactive ligand-receptor interaction and G protein-coupled receptor signaling pathway. GSEA enrichment analysis indicated that ALG3 may be regulated with “MYC targets, E2F targets, DNA repair, G2M checkpoint, and MTORC1 signaling”. Disorders in the MYC expression are not conducive to patient survival and are involved in cell proliferation, apoptosis, differentiation, and metabolism (Chen et al., 2018). Increased E2F activity led to an abnormal cell cycle (Kent and Leone, 2019). Considering our results, we could predict that ALG3 may participate in crucial biological processes.

In addition, the tumor microenvironment has been confirmed to play an important role in tumorigenesis and progression of immune cells and is a key role in this process (Hinshaw and Shevde, 2019). Therefore, we used TIMER2.0 and CIBERSORT to assess the relationship between ALG3 and immune cell infiltration in LIHC. The result indicated that ALG3 expression was highly related to the enrichment of TILs, including B cells, CD8^+^ T cells, dendritic cells, neutrophils, CD4^+^ T cells, and macrophages in HCC. It has been reported that the interaction between tumor-infiltrating B cells and T cells promotes the progression of HCC (Garnelo et al., 2017). Single-cell analysis of primary HCC and recurrent HCC showed that CD8^+^ T cells and DCs were increased in relapsed HCC, and the aberrant expression led to the compromised antitumor immunity (Zhang et al., 2019). Based on our studies, we could speculate that ALG3 may participate in the occurrence and development of liver cancer by regulating and recruiting the expression of immuno-infiltrating cells.

In order to further explore whether ALG3 affects the biological behavior of HCC cells, we have established transient cell lines. As shown in Figure 7B, CCK-8 and MTT results showed that ALG3 had no effect on the proliferation of HCC cells. However, the results of the transwell assay showed that knockdown of ALG3 inhibited migration in Hep3B (Figure 7C). Consistent with the results of previous studies, two inferences can be drawn as follows. First, ALG3 may only affect the survival prognosis of patients by affecting the invasion of HCC cells, and the effect on the proliferation of HCC cells is not obvious. Second, a previous study has reported that ALG3 can affect the proliferation of BRCA, OSCC, and NSCLC (Shao et al., 2021; Ke et al., 2020; Sun et al., 2021), indicating that ALG3 can affect the proliferation of some cancer cells. In Figure 7A, only Hep3B was successfully constructed among the three cell lines, suggesting that the expected results could not be achieved due to the lack of quality of si-ALG3. In addition, researchers can now assess the glycoprotein secreted on the cell surface to reflect the overall cellular state in health and disease. It has been suggested that altered glycosylation of proteins that participate in the immunological synapse affects the signaling processes and cell proliferation, as well as exacerbation of the effector mechanisms of T cells that trigger systemic damage and autoimmunity (Gómez-Henao et al., 2021). Current research indicates that changes in some glycosylated proteins can cause the immune escape of viruses and promote metastasis of cancer cells, but some glycosylated proteins can also regulate the cell cycle of cancer cells and induce apoptosis of tumor cells (Reily et al., 2019). New insights into the structure and function of glycosylates can be applied to therapeutic development, improving our ability to regulate immune response and inflammation. Studies on the correlation between glycosylation and the regulation of immune response (Li et al., 2018), PD-1 receptor pathway (Liu et al., 2020b), and tumor microenvironment (Chandler et al., 2019) suggest that new tumor therapeutic targets may be found by paying attention to glycosylation, so we planned to construct stable cell lines to continue to study deeper mechanisms and explore the possible pathways of ALG3 affecting hepatocellular carcinoma.

To sum up, our findings proved that ALG3 overexpression resulted in shorter overall survival in HCC patients and may promote tumor progression and immune cell infiltration. Collectively, our study provided a potential independent unfavorable prognostic biomarker for HCC patients and new insights into the mechanism of HCC tumorigenesis and progression.

## Data Availability

The original contributions presented in the study are included in the article/Supplementary Material; further inquiries can be directed to the corresponding authors.
