# Supplementary material for: Expression of ALG3 in Hepatocellular Carcinoma and Its Clinical Implication
Source: Front Mol Biosci. 2022 Jun 15;9:816102. doi: 10.3389/fmolb.2022.816102 (PMC9240429; doi:10.3389/fmolb.2022.816102)
Supplement: Supplementary file 1 [file Table1.DOCX]

**Supplementary Table 1:** Characteristics of hepatocelular cancer patients

| Characteristics | No. of patients (%) |
| --- | --- |
| Age(years) |  |
| ≤60 | 60(52) |
| >60 | 55(48) |
| Gender |  |
| male | 79(69) |
| female | 36(31) |
| Histology classification |  |
| Ⅰ-Ⅱ | 40(35) |
| Ⅲ-Ⅳ | 75(65) |
| TNM stage(AJCC) |  |
| T1-T2 | 89(77) |
| T3-T4 | 26(23) |
| Tumor size(cm) |  |
| <5 | 43(37) |
| ≥5 | 72(63) |
| Tumor number |  |
| single | 90(78) |
| multiple | 25(22) |
| Tumor capsular |  |
| complete | 96(83) |
| imcomplete | 19(17) |
| Cut edge |  |
| negative | 107(93) |
| positive | 8(7) |
| Vascular tumor emboli |  |
| present | 45(39) |
| absent | 70(61) |
| Vital status |  |
| Alive | 51(44) |
| Dead | 64(56) |
| Recurrent status |  |
| Yes | 79(69) |
| no | 36(31) |
| Serum AFP (ng/ml) |  |
| <400 | 76(66) |
| ≥400 | 39(34) |
| HBVDNA |  |
| <500 | 79(69) |
| ≥500 | 36(31) |
| Tumor location |  |
| right lobe | 69(60) |
| left lobe | 35(30) |
| middle Lobe | 6(5) |
| others | 4(4) |
| multiple | 1(1) |
| HBsAg |  |
| negative | 41(36) |
| positive | 74(64) |
| HbeAg |  |
| negative | 99(86) |
| positive | 16(14) |
| HbcAb |  |
| negative | 49(43) |
| positive | 66(57) |
| HCV |  |
| negative | 106(92) |
| positive | 9(8) |
| Modus Operandi |  |
| open | 84(73) |
| laparoscopic | 28(24) |
| other | 3(3) |
| Anatomical resection |  |
| Yes | 28(24) |
| No | 87(76） |
| Intraoperatve blood loss |  |
| ≤400 | 67(58) |
| >400 | 48(42) |
| Intraoperative blood transfusion |  |
| no | 89(77) |
| yes | 26(23) |
| Postoperative complication |  |
| No | 102(89) |
| postoperation hemorrhage | 4(3) |
| bile leakage | 3(3) |
| liver failure | 1(1) |
| others | 5(4) |
| Postoperative adjuvant therapy |  |
| hyperthermic intraperitoneal perfusion | 8(6) |
| TACE | 39(34) |
| Sorafenib | 3(3) |
| no | 65(57) |

**Supplementary Table 2:** Gene primers

| Gene Name | Forward primer | Reverse primer |
| --- | --- | --- |
| ALG3 | CACCGTTAAGATGGCGGCT | CCATTGCTTGCAGAGTCCCT |
| GAPDH | GTCTCCTCTGACTTCAACAGCG | ACCACCCTGTTGCTGTAGCCAA |
| β-actin | CTCCATCCTGGCCTCGCTGT | ACTAAGTCATAGTCCGCCTAGA |

**Supplementary Table 3:** Dataset of ALG3 differential expression

| Datasets | number of patients | pvalue |
| --- | --- | --- |
| GSE25097 |  |  |
| HCC | 268 | <0.01 |
| adjacent | 243 |  |
| GSE36376 |  |  |
| HCC | 240 | <0.01 |
| adjacent | 193 |  |
| ICGC-LIRI-JR |  |  |
| HCC | 212 | <0.01 |
| adjacent | 177 |  |
